# Well-Differentiated Liposarcoma of the Spermatic Cord Involving Testicular Parenchyma

**DOI:** 10.14740/jmc5346

**Published:** 2026-09-04

**Authors:** Allen Barbarovich, Sean Higgins, James Chambers

**Affiliations:** a Department of Internal Medicine, Northeast Georgia Medical Center, Gainesville, GA 30501, USA; b Department of General Surgery, Northeast Georgia Medical Center, Gainesville, GA 30501, USA

**Keywords:** Liposarcoma, Spermatic cord, Orchiectomy, Para-testicular neoplasm

## Abstract

Liposarcomas of the spermatic cord are rare malignant soft-tissue tumors, representing a small fraction of adult sarcomas and an even smaller proportion of para-testicular neoplasms. These tumors often present as slow-growing, painless inguinoscrotal masses and may be mistaken for benign conditions such as inguinal hernias or lipomas. We present the case of a man in his mid-60s who presented with a progressively enlarging right hemi-scrotal mass. Imaging revealed a large fat-containing lesion involving both the spermatic cord and testicular parenchyma, suspicious for fat-containing hernia. The patient underwent right radical inguinal orchiectomy with high ligation of the cord. Histopathologic evaluation demonstrated a well-differentiated liposarcoma without lymphovascular or perineural invasion, with negative surgical margins and no evidence of dedifferentiation. The tumor measured approximately 18 cm in total, involving both cord and testicular tissue. The post-operative course was uneventful, and surveillance imaging has shown no recurrence to date. This case highlights the importance of maintaining a high index of suspicion for malignant etiologies in atypical scrotal masses, the role of radical orchiectomy with wide resection in management, and the necessity for long-term follow-up given the potential for late recurrence even in well-differentiated tumors.

## Introduction

Soft-tissue sarcomas are rare, comprising approximately 1% of adult malignancies, with liposarcomas accounting for around 20% of these cases [1]. Spermatic cord liposarcomas are even rarer, representing only 3–7% of all liposarcomas, and 7–10% of intra-scrotal tumors [2]. Among intra-scrotal liposarcomas, the spermatic cord is the most common site (∼76%), followed by testicular tunics (20%) and epididymis (4%) [2]. These tumors typically are present in middle-aged to older adults (50–60 years), often as painless, slowly enlarging inguinoscrotal masses frequently misdiagnosed as inguinal hernias, lipomas, hydroceles, or seromas [3]. Imaging such as ultrasound, computed tomography (CT), and magnetic resonance imaging (MRI) are vital for pre-operative assessment. Characteristic imaging findings include a predominantly fat-containing mass with thick septations (> 2 mm), nodular non-adipose components, internal enhancement, heterogeneous attenuation, and progressive enlargement. MRI may further demonstrate T1 hyperintensity with incomplete fat suppression and enhancing septa or nodules [4]. Histologic classification follows the World Health Organization (WHO) guidelines: well-differentiated (WDLPS), dedifferentiated (DDLPS), myxoid, round-cell, and pleomorphic types [5]. Histologically, WDLPS is characterized by mature adipocytes demonstrating marked variation in cell size, fibrous septa containing atypical hyperchromatic stromal cells, scattered lipoblasts, and variable degrees of nuclear pleomorphism. Amplification or overexpression of MDM2 and CDK4 helps distinguish WDLPS from benign lipomatous tumors. The mainstay of treatment remains radical inguinal orchiectomy with wide local resection and high ligation of the cord [2]. The role of adjuvant radiotherapy is controversial but may lower local recurrence; chemotherapy efficacy is limited and generally reserved for high-grade or dedifferentiated tumors [6, 7]. Long-term follow-up is strongly recommended due to notable rates of local recurrence up to 10% even many years post-surgery [8]. According to the National Comprehensive Cancer guidelines, the recommendation includes a physical exam with imaging every 3–6 months for the first 3 years, every 6 months to complete 5 years, and then annually in perpetuity [8–10].

## Case Report

### Investigations

A 65-year-old man presented with a progressively enlarging right hemi-scrotal mass. The mass was painless, with no associated systemic symptoms. Physical examination revealed a firm, non-tender scrotal mass separate from the contralateral testis.

### Diagnosis

**Figure 1:**
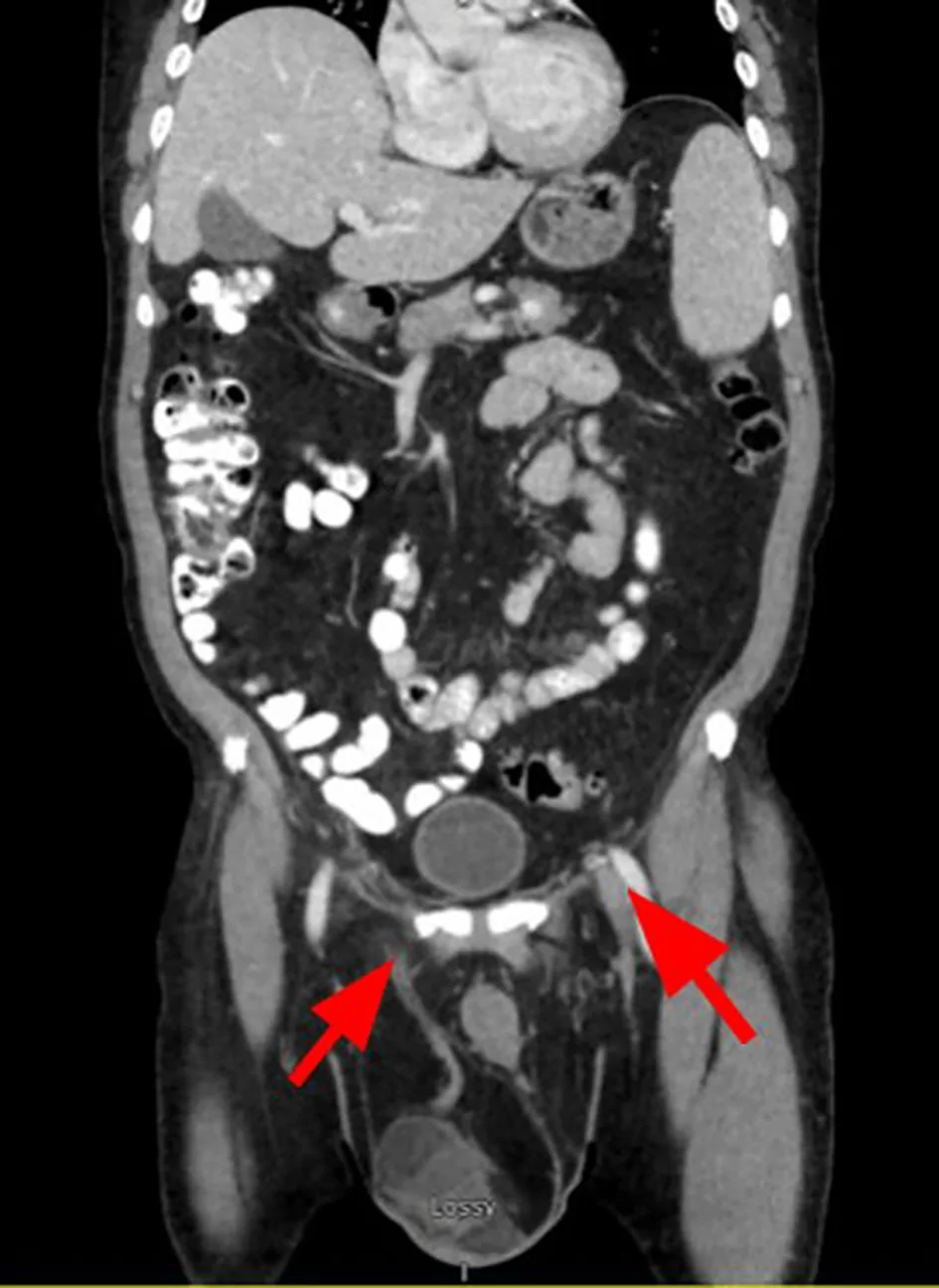
Coronal contrast-enhanced CT of the abdomen and pelvis demonstrating bilateral fat-containing inguinal masses (red arrows). The larger right-sided lesion extends through the inguinal canal into the right hemiscrotum and was initially interpreted as an inguinal hernia containing mesenteric fat. Retrospective review demonstrated internal soft tissue nodularity and mass effect concerning liposarcoma. CT: computed tomography.

Scrotal ultrasound and CT revealed a large fat-containing mass involving the spermatic cord and extending into the testicular parenchyma, suspicious for hernia with peritoneal fat contents (Fig. 1). Differential diagnoses included inguinal hernia, lipoma, atypical lipomatous tumor/WDLPS, and other paratesticular sarcomas. Although the lesion was initially interpreted as a fat-containing inguinal hernia, a retrospective review identified imaging features concerning WDLPS, including large size, heterogeneous fat attenuation, internal septations, and extension along the spermatic cord.

### Treatment

A right-sided inguinal incision was extended towards the superficial inguinal ring, after which blunt dissection was carried down through the external abdominal oblique sheath transversely from the superficial ring, to just lateral of the deep inguinal ring to fully expose the spermatic cord.

Upon exposure, the cord was lifted from the inguinal canal, and a Penrose drain was then used to isolate the cord lateral to the inferior epigastric vessels. The cord was then dissected into separate bundles including vas deferens, blood vessels and hernial sac, and carefully examined for the presence of bowel. Multiple 0 vicryl free ties and suture ties were placed for complete separation of these bundles, which were then incised to fully isolate the cord and reflect it outside of the inguinal canal.

Dissection was then continued towards the scrotum, which was carefully dissected circumferentially without any disruption of the enclosing layers, and finally towards the distal portion where the testicle was separated from the scrotum. The right orchiectomy specimen with attached spermatic cord and lobulated yellow-tan fatty mass was removed. The specimen overall weighed 505 g, and the attached spermatic cord measured 11.5 cm in length and up to 4.5 cm in diameter. Attached lobulated fatty mass was present at the junction of the spermatic cord and testicle. The outer portion of the testicular resection was gray-tan and smooth. The testicle was bivalved, revealing a lobulated yellow-tan to tan-brown mass, 9.0 × 7.0 × 6.5 cm. The previously described portion of lobulated fatty tissue at the junction of the spermatic cord and testis appeared to be a portion of this mass. The testicular tissue measured 5.0 × 4.5 × 3.5 cm. Testicular tissue appeared compressed without evidence of gross infiltration by the fatty tissue. The margin of the fatty tissue external to the testis was inked. The epididymis was identified and appeared grossly uninvolved. No gross intratesticular mass was identified.

Both the scrotal side and the inguinal canal were carefully inspected for bleeding; hemostasis was fully secured prior to proceeding to closing the abdominal wall defect. The defect identified was small, and the decision was made to place an 11 × 5 cm cone-shaped cut piece of ProGrip mesh on the inguinal floor, affixed at four points with 0 Ethibond sutures. The external oblique anastomosis was closed over the mesh with a running 2-0 vicryl. The Scarpas fascia was closed with interrupted 3-0 vicryl sutures. The skin was approximated with a running 3-0 Monocryl and glue. The patient underwent right radical inguinal orchiectomy with high ligation of the spermatic cord and en-bloc removal of surrounding soft tissue. Gross pathology revealed a combined tumor size of approximately 18 cm. The patient’s post-operative recovery was uneventful.

### Follow-up and outcomes

At 1-month follow-up, there was evidence of seroma formation, not unexpected given the original mass effect of the tumor, but no evidence of recurrence. Regular follow-up for the next 6 months was recommended to evaluate the progression of seroma, and long-term follow-up surveillance was recommended given the risk of late recurrence. Ambulatory referral to medical oncology was also issued to discuss any indication for adjuvant therapies.

**Figure 2:**
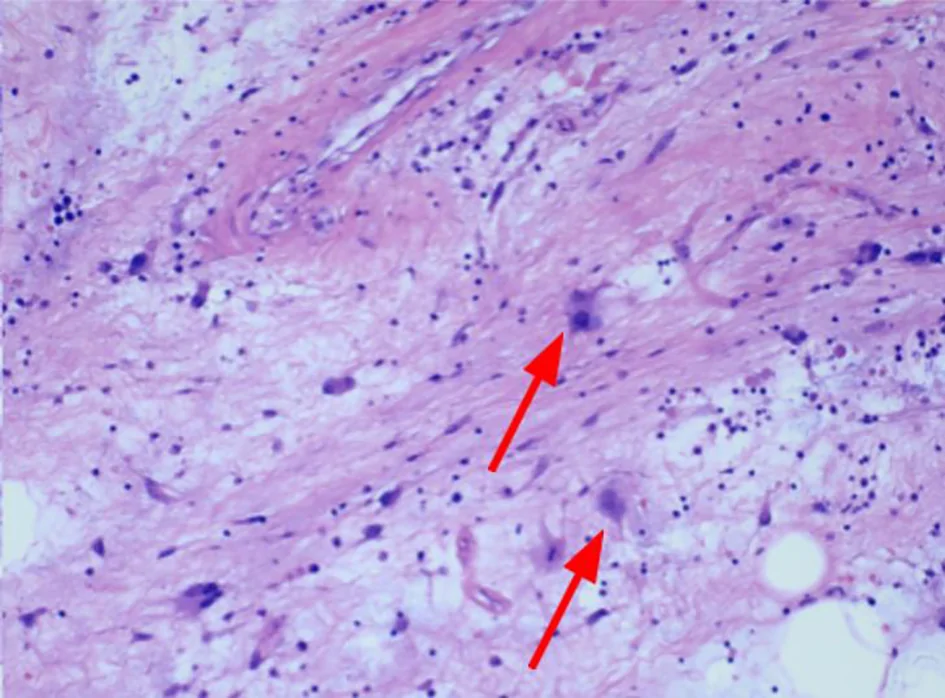
Hematoxylin and eosin stain slides of excised scrotal mass. The tumor is composed of adipocytes with bands of fibrotic stroma showing markedly atypical cells with enlarged, hyperchromatic nuclei (red arrows).

Histology demonstrates WDLPS with no lymphovascular or perineural invasion (Fig. 2). Immunohistochemistry demonstrated nuclear positivity for MDM2 and CDK4, supporting the diagnosis of WDLPS. Margins are negative for neoplastic cells and no overt evidence of dedifferentiation is seen. The tumor is present as a proliferation within the spermatic cord (9.0 cm), as well as present in the testicular parenchyma (9.0 cm), with a combined size of approximately 18 cm.

## Discussion

This case's involvement of both the spermatic cord and testicular parenchyma in a combined 18-cm tumor is rare—most paratesticular liposarcomas exceed 10 cm only in a handful of giant paratesticular liposarcoma case reports [11, 12]. Several features distinguish this case from previously reported spermatic cord liposarcomas. First, the tumor demonstrated apparent extension into the testicular parenchyma, an uncommon finding because most spermatic cord liposarcomas remain confined to paratesticular tissues. Second, the combined tumor size of approximately 18 cm places it among the larger cases reported in the literature. Third, the lesion was initially interpreted as a bilateral fat-containing inguinal hernia, highlighting the ongoing diagnostic challenge posed by these tumors. These tumors often mimic benign pathologies such as hernias or lipomas, leading to misdiagnosis or delayed diagnosis. In fact, 87% of paratesticular sarcomas are referred post-operatively, and only 43% were imaged pre-operatively [1, 13, 14]. Up to 24% required reresection due to incomplete initial excision performed under presumed initially benign diagnosis [13]. Furthermore, in a 2024 meta-analysis, 68% of paratesticular sarcomas required revision secondary to initial misdiagnosis as a hernia, lipoma, or a hydrocele. Among liposarcomas, 12.5% had been previously resected “lipomas,” and 31% of cases had residual tumor without negative borders at revision [14]. All adipose from hernia repairs greater than 10 cm are recommended for referral to histological evaluation [15]. Advanced imaging (CT, MRI) is critical for accurate pre-operative assessment, but remains often unused as the standard workup for an otherwise non-suspicious hernia on physical exam is clinical, and there is little indication for advanced imaging without suspicion of complicated anatomy [3, 4]. MRI was not performed because the lesion was initially interpreted as a large inguinal hernia containing fat based on ultrasound and CT findings. Given the diagnostic uncertainty highlighted by this case, MRI may be valuable in patients with atypical fat-containing inguinoscrotal masses, as it can better characterize soft tissue composition and identify imaging features suggestive of liposarcoma. Pre-operative biopsy was not pursued because imaging findings were considered most consistent with a benign inguinal hernia. Additionally, when spermatic cord sarcoma is strongly suspected, concerns regarding biopsy tract contamination and the need for definitive en-bloc resection often favor upfront radical orchiectomy. Radical orchiectomy with wide cord excision remains the gold standard, aiming for negative margins and optimal local control [2, 8]. In giant or complex tumors, hemiscrotectomy may also be required [12]. Achieving completely negative margins, as in our case, likely contributes to favorable prognosis. Negative margins have a 3-year local recurrence-free survival rate of 100% versus 29% for positive margins (P = 0.0005) [3, 8]. Margins were a strong predictor of local recurrence even with adjunct radiation [7, 8, 16]. Wide reresection was associated with recurrence-free survival (P < 0.0001), and positive margins remain associated strongly with both recurrence and death (P = 0.034 and P = 0.025, respectively) [8, 16]. For well-differentiated tumors, adjuvant radiotherapy may not be necessary. However, in cases of close margins or dedifferentiation, radiotherapy can reduce recurrence risk [7]. Adjuvant radiotherapy may be considered in patients with positive or close margins, recurrent disease, or dedifferentiated histology. Chemotherapy has limited efficacy in WDLPS but may be considered for unresectable, recurrent, metastatic, or dedifferentiated tumors. Emerging systemic therapies targeting MDM2 and CDK4 are under investigation but are not currently standard of care. Chemotherapy is more relevant in high-grade or dedifferentiated subtypes, though responses are limited [6]. WDLPS generally portend a favorable prognosis, especially when margins are negative and no dedifferentiation is evident [17]. Nevertheless, the 10% recurrence rate, often after several years, dictates the need for long-term surveillance [3, 8]. This case underscores the necessity of suspicion for malignancy in slow-growing scrotal masses, the importance of comprehensive pre-operative imaging, and the surgical imperative of wide excision.

### Conclusions

WDLPS of the spermatic cord and testis is a rare entity that can mimic benign scrotal pathology. This case highlights key teaching points: maintain suspicion for malignancy in enlarging or atypical scrotal masses; prioritize complete surgical resection with high-cord ligation and negative margins as the cornerstone of management; and recognize that, in the absence of high-risk features, prognosis is generally favorable without routine need for adjuvant therapy. Given the risk of late local recurrence, diligent long-term surveillance is essential, incorporating periodic imaging, regular clinical follow-up, and patient education, with a low threshold for reassessment if new symptoms arise.

## Data Availability

All data generated or analyzed in the report are included in this published article. Additional information is available from the corresponding author upon reasonable request.
